# Erosive Potential of Bottled Salad Dressings

**DOI:** 10.3290/j.ohpd.b898955

**Published:** 2021-01-26

**Authors:** Julia J. Hartz, Alessio Procopio, Thomas Attin, Florian J. Wegehaupt

**Affiliations:** a Resident, Clinic of Conservative and Preventive Dentistry, Center of Dental Medicine, University of Zürich, Zürich, Switzerland. Wrote the manuscript.; b Resident, Clinic of Conservative and Preventive Dentistry, Center of Dental Medicine, University of Zürich, Zürich, Switzerland. Performed the experiments in partial fulfilment of requirements for a master degree. *Shared first authorship.; c Professor and Director, Clinic of Preventive Dentistry, Periodontology and Cariology, Center of Dental Medicine, University of Zürich, Zürich, Switzerland. Research idea, contributed substantially to discussion, proofread the manuscript.; d Head of Division of Preventive Dentistry and Oral Epidemiology, Clinic of Preventive Dentistry, Periodontology and Cariology, Center of Dental Medicine, University of Zürich, Zürich, Switzerland. Research idea, hypothesis, experimental design, contributed substantially to discussion and writing the paper, proofread the manuscript.

**Keywords:** erosion, tooth wear, salad dressings

## Abstract

**Purpose:**

A previous clinical study showed that the prevalence of erosive toothwear in vegetarians is statistically significantly higher than in nonvegetarians, due to the consumption of vinegar and other acidic foodstuffs. To adequately inform patients, this study investigated the erosive potential of bottled salad dressings available in Switzerland and compared it with that of orange juice.

**Materials and Methods:**

One hundred enamel samples of bovine teeth were divided into ten groups. Samples were placed in 1 of 9 bottled salad dressings or orange juice (Granini) for 2 min. Afterwards, they were rinsed with Zürich tap water for 30 s, followed by abrasion with a toothbrush for 20 brush strokes and a toothpaste-saliva mixture. Erosive/abrasive enamel wear was determined with contact profilometry after 40 cycles.

**Results:**

The enamel wear (median/IQR) caused by Tradition Sauce Balsamique (9.5 µm/5.3 µm), M-Classic Dressing Italiano (10.9 µm/12.3 µm), Betty Bossi Balsamico Dressing (9.4 µm/4.5 µm) and Thomy Balsamico Vinaigrette Dressing (14.2 µm/6.5 µm) was statistically significantly higher than that caused by orange juice (2.4 µm/0.8 µm). Enamel wear caused by M-Classic Dressing French Joghurt (0.2 µm/0.2 µm) and Coop Qualité & Prix French Dressing (1.2 µm/1.0 µm) was statistically significantly lower compared to that of orange juice.

**Conclusions:**

The pure balsamico vinegar-based dressings (Italian type) showed a statistically significantly higher erosive potential than orange juice, whereas dressings containing calcium-rich products (enriched with milk and/or cream) (French-type) caused lower enamel wear than orange juice. The study shows that some bottled dressings have erosive potential even higher than orange juice and patients should be informed accordingly.

Erosion is a chemically induced, irreversible loss of dental hard tissue by endogenous or exogenous acids not produced by microorganisms.^1,13,40,49^ Intrinsic erosion is caused by gastric juice (hydrochloric acid) entering the oral cavity.^16,18,33,41^ However, erosion is mainly caused by the consumption of extrinsic acids. These are found in foods/beverages such as citrus fruits, salad dressings, soft drinks and alcoholic beverages.^17,30,40^

In the initial phase of the erosive process, the protons released by the acids react with the carbonate or phosphate of the enamel apatite, thus destabilising the enamel crystals and causing the dissolution of dental minerals.^30^ Unchecked erosion can ultimately reach the dentin. This irreversible loss of substance is accelerated when the tooth surface is exposed to additional abrasive processes (for example toothbrushing).^4^ Undersaturation of the acid solution with respect to dental hard tissue is a prerequisite for the removal of ions from the enamel. In contrast, saliva is an ion-supersaturated solution and can remineralise softened tooth substance.^39^

The type, frequency and duration of acid exposure are crucial for the development of erosions.^32^ However, several other factors such as acid strength, temperature and the concentration of phosphate, calcium and fluoride ions also determine the erosive potential of an acid.^15,27^ Other erosion-modifying and individual factors are salivary calcium concentration, the interaction with the enamel cuticle,^9^ the salivary flow rate and the buffering capacity of the saliva.^11^

There is evidence that the prevalence of erosion is increasing continuously. The growing consumption of highly acidic foods has increased the risk of dental erosion in recent years. Not only greater consumption of soft drinks,^12,29,34^ but also the increasing number of vegetarians, who consume significantly more fruits and vegetables^31^ and accordingly more vinegar in salads,^23^ can be assumed.

However, a recent review showed that prevalence data of dental erosion are not homogeneous.^20^ It is difficult to compare results of epidemiological studies due to different examination standards (scoring systems, samples and examiners) and variety of groups examined (grouped by age, gender, or geographical location). The review^20^ summarised that 1% to 79% of children between 2 and 5 years of age had erosions on their deciduous teeth. 14% of children between the ages of 5 and 9 already had erosive damage on their permanent teeth. The prevalence was between 7% and 100% for children and adolescents between 9 and 20 years and between 4% and 100% for adults between 18 and 88 years.^20^

However, a previous study reported that lactovegetarians showed more erosive defects than non-lactovegetarians.^23^ Of the examined lactovegetarians, 26.9% showed already incipient, 19.2% moderate and 30.8% severe erosive defects. No erosion was observed in the control group (non-lactovegetarians). The increased prevalence of erosion among lactovegetarians has been associated with the consumption of vinegar, pickles, citrus fruits and acidic berries. It is assumed that vinegar is not consumed solely, but as an ingredient of salad dressings.

Thus, aim of the present study was to investigate the erosive potential of bottled salad dressings in comparison with orange juice. The null hypothesis was that there is no significant difference in enamel loss of different bottled salad dressings.

## Materials and Methods

### Sample Preparation and Allocation

Enamel samples were prepared from bovine mandibular anterior teeth acquired from a local slaughterhouse (SBZ Schlachtbetrieb Zürich; Zürich, Switzerland). A total of 100 enamel samples were obtained using a diamond hollow drill with an internal diameter of 3 mm. The samples were marked according to the tooth from which they originated, for use during later sample allocation. The enamel samples were embedded in resin (Paladur Elite, Heraeus Kulzer; Hanau, Deutschland) which was was polymerised in a pressure cooker (45°, 4.8 bar) for 12 min. After embedding, the enamel/resin samples had an outer diameter of 6 mm and were ground with a water-cooled grinding machine (1000, 2000, 4000 grit) (GEKO SiC Foil, Struers; Ballerup, Denmark) to achieve a flat surface. Two parallel reference scratches 2 mm apart were made in the resin and enamel, for later profilometric measurement as described in previous studies.^44^ Finally, samples were randomly divided into ten groups (G1–G10, n = 10). Care was taken that each group did not contain more than one sample from any single tooth. Until further use, samples were stored in Zürich tap water (mean pH: 7.87; mean fluoride content: 0.06 mg/l).^50^

### Erosive Solutions (Bottled Salad Dressings and Orange Juice)

For this study, bottled salad dressings were selected from Coop and Migros, the two largest retailers in Switzerland. A distinction was made between balsamic vinegar-based dressings (Italian style) and dressings containing milk and/or cream (French style) (Table 1). Granini orange juice served as a positive control.

**Table 1:** Test liquids: ingredients of bottled salad dressings and orange juice

Product	Ingredients according to manufacturer
Anna’s Best Dressing Balsamico*	Water, Aceto balsamico di Modena, I.G.P. 30%, (red wine vinegar, grape must, colouring agent: E 150d), olive oil, sunflower oil, sugar, concentrated grape juice, seasoning, saline, natural flavourings, thickener (E 415)
Betty Bossi Balsamico Dressing**	Water, Aceto Balsamico di Modena I.G.P. 18% (wine vinegar, grape must, colouring agent: E 150d), rapeseed oil, olive oil, sugar, sea salt, onions, parsnip purée, basil, dye (E 150d), garlic, thickener (E 415), pepper, nutmeg
M-Classic Dressing Italiano*	Water, red wine vinegar 37%, olive oil 7%, sugar, sunflower oil, table salt, spices (maltodextrin, sugar, oregano, parsley, chives, red pepper, garlic, pepper, table salt, sunflower oil, flavour enhancer: E 621), onion, elderberry concentrate, natural aromas, basil, parsley, thickeners: E 415 and E 401
Thomy Balsamico Vinaigrette Dressing***	Water, Aceto Balsamico 25% (wine vinegar, grape must, colouring agent E150d and antioxidant E220), sunflower oil 15%, table vinegar, olive oil “extra vergine” 5%, iodised table salt, flavour enhancer (sodium glutamate), thickening agents (guar gum, xanthan gum), Parsley, spice extracts. common salt, total content: 3.1 g / 100ml
Thomy Classics Dressing Italian***	Water, red wine vinegar 29%, sunflower oil, iodised table salt, sugar, flavour enhancer (monosodium glutamate), antioxidant (potash30 umcitrate), thickening agent (carrageenan, xanthan gum, locust bean gum), garlic, yeast extract, dried onion, spice, aroma, herbs, elderberry juice, caramelised sugar
Tradition Sauce Balsamique*	Sunflower oil, Aceto balsamico di Modena 30% (wine vinegar, concentrated grape must),water, burnt sugar, table salt, concentrated lemon juice, yeast extract, garlic, spice, onion, thickener: E 415, basil, natural flavour (contains celery), pepper
Coop Qualité & Prix French Dressing**	Water, rapeseed oil, herbal table vinegar (table vinegar, herbal extract, iodised table salt), mustard (water, mustard seeds, table vinegar, sugar, spices), seasoning (table salt, flavour enhancer [E 621], sugar, onions, palm oil, yeast extract, potato starch, flavour, spices), egg yolk (from barn eggs), onions, iodised table salt, herbs 0.6%, sugar, parsnip purée, tomato flakes, thickener (E 415), nutmeg
M-Classic Dressing French*	Water, sunflower oil, alcohol vinegar, mustard, onion, egg yolk (from barn eggs), table salt, bouillon, yeast extract, thickeners: E 415, parsley, natural flavours
M-Classic Dressing French Joghu*	Water, semi-skimmed yoghurt 30% (skimmed milk, cream, milk proteins), sunflower oil, alcohol vinegar, sugar, white wine vinegar, table salt, egg yolk (from barn eggs), seasoning (flavor enhancer: monosodium glutamate), garlic, thickener: E 415, mustard, aromas, natural flavours, pepper extract, emulsifier: soya lecithin
Granini orange juice (made for Eckes-Granini Suisse; Henniez, Switzerland)	100% orange juice from concentrate, pasteurised. Without pulp

*Made for Migros cooperative, Zürich, Switzerland; **made for Coop cooperative, Basel, Switzerland; ***made for Nestlé Suisse SA, Vevey, Switzerland.

The ingredients of the tested dressings are listed in Table 1.

### Characterisation of the Solutions

The calcium content of the solutions was determined by atomic absorption spectrometry (ContrAA 300, Analytik Jena; Überlingen, Deutschland) in the aqueous phase.

To determine pH values, the dressings were centrifuged at 4000 rpm for 10 min to separate the oil and aqueous phases. The pH values of the products used in this study were subsequently measured using a pH electrode at 22°C (Metrohm E 632, Metrohm Switzerland; Herisau, Switzerland) in the aqueous phase. The pH values and calcium contents of the salad dressings and the orange juice are presented in Table 2.

**Table 2:** Test liquids: Selection of bottled salad dressings and orange juice with their manufacturers, pH value and calcium content

Group	Product	pH-value	Calcium-content (mmol/l)	Dressing type
G1	Anna’s Best Dressing Balsamico*	3.33	4.90	Italian
G2	Betty Bossi Balsamico Dressing**	3.23	4.76	Italian
G3	M-Classic Dressing Italiano*	3.27	2.90	Italian
G4	Thomy Balsamico Vinaigrette Dressing***	3.64	3.21	Italian
G5	Thomy Classics Dressing Italian***	3.88	4.81	Italian
G6	Tradition Sauce Balsamique*	3.12	5.27	Italian
G7	Coop Qualité & Prix French Dressing**	3.57	5.81	French
G8	M-Classic Dressing French*	3.87	4.02	French
G9	M-Classic Dressing French Joghu*	4.07	15.23	French
G10	Granini orange juice (made for Eckes-Granini Suisse; Henniez, Switzerland)	3.76	1.86	Control product

*Made for Migros cooperative, Zürich, Switzerland; **made for Coop cooperative, Basel, Switzerland; ***made for Nestlé Suisse SA, Vevey, Switzerland.

### Erosive/Abrasive Procedure

Before starting the erosive/abrasive procedure, reference areas (areas outside the two parallel scratches applied to enamel and resin) were covered with adhesive tape leaving a test area in between the reference areas uncovered.

To perform the erosive attack, samples were immersed in the respective dressing or orange juice for 2 min. Each sample was eroded with 3 ml of the group-specific solution. The solutions were kept in motion by gently shaking the container with the samples and the solution. Afterwards, the samples were rinsed with Zürich tap water for 30 s to remove remnants of the solutions. Next, toothbrush abrasion was performed in an automatic brushing machine^19^ for 20 brushing strokes per sample. For brushing, a manual-toothbrush head was used (paro M 43, Esro; Kilchberg, Switzerland) with a constant brushing load of 2 N. During brushing, the samples were covered with a toothpaste slurry prepared by mixing 200 ml artificial saliva^21^ and 100 ml toothpaste (ELMEX caries protection toothpaste; Gaba Colgate Palmolive; Therwil, Switzerland). After brushing, the samples were removed from the brushing machine and rinsed off with tap water. One erosive/abrasive cycle consisted of one erosive attack and one abrasive attack as described above. For each erosive and abrasive attack, fresh solutions and slurry were used, respectively.

A total of 40 erosive/abrasive cycles were performed on each enamel specimen.

### Determination of Enamel Wear

Enamel wear was determined by contact profilometry (MarSurf GD25, Mahr; Göttingen, Germany). In this process, a fine needle scans the surface of the sample and creates a height profile. Five profiles with a set distance of 250 μm between each profile per sample were recorded before (baseline profile) and after 40 cycles (final profile). The previously made scratches and protected reference areas on each sample enabled exact superimposition of the baseline with the respective final profiles. If the wear per profile was below the profilometer’s measurement limit of 0.105 μm,^2^ the value for this profil was set as 0.000 μm.

Per sample, the tooth wear was calculated by averaging the values of the five respective profiles. Per group, the respective values were calculated by averaging the values of the 10 samples of the respective group.

### Statistical Analysis

The statistical evaluation was performed using the Kruskal-Wallis rank sum test for independence of the data. In addition, the data were compared pairwise with the Conover post-hoc test, which compares multiple independent data with each other. The significance level was set at p ≤ 0.05.

All statistical work was conducted with the statistical software R (R Foundation for Statistical Computing; Vienna, Austria).

## Results

**Fig 1:**
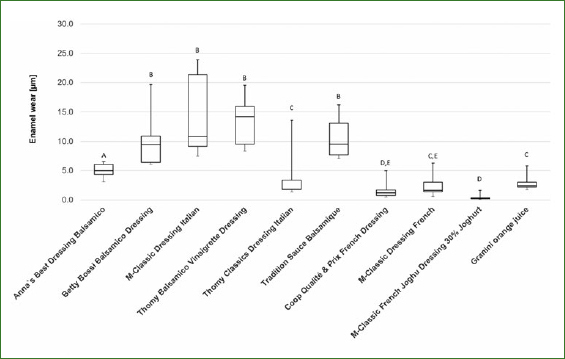
Enamel wear (median and interquartile range, IQR = whiskers) for the salad dressings and orange juice after 40 erosive/abrasive cycles. Values which did not differ from each other at the 5% level are designated with the same capital letters.

Enamel wear after 40 cycles of erosion and abrasion for the different groups is illustrated in Fig 1. The median enamel wear of Granini orange juice was 2.4 µm.

All balsamic vinegar-based dressings except Thomy Classics Dressing Italian showed statistically significantly higher enamel loss than did orange juice (p < 0.05, respectively). The enamel wear caused by Thomy Classics Dressing Italian did not differ statistically significantly from that caused by orange juice (p > 0.05).

The French-type dressings, containing milk and/or cream, M-Classic Dressing French Joghu and Coop Qualité & Prix French Dressing, caused statistically significantly lower enamel wear than did orange juice (p < 0.05). The enamel wear for M-Classic Dressing French showed no statistically significant difference to that of orange juice (p > 0.05).

## Discussion

In this study, 2-min erosion was performed per sample, followed by abrasion with 20 brushing strokes. This corresponds to the study design commonly used in erosion and erosion/abrasion studies (≤ 2 min erosion, 10–15 brush strokes with a force of 1–2 N).^47^

For this study, enamel samples were obtained from bovine teeth. They are quite easy to obtain in large numbers in slaughterhouses and also have a fairly large surface area, so that more than one sample per tooth can be obtained.^47^ It can be assumed that bovine teeth do not have a history of fluoride measures like human teeth do, so that a possible influence of different fluoride treatments on the erosive procedure can be ruled out. Therefore, the homogeneity of the bovine samples obtained should be considered as a further advantage over human teeth.^48^

However, due to different genetic, environmental and dietary influences, there are differences in the physicochemical properties and morphology of human and bovine teeth.^22^ Bovine enamel is more susceptible to demineralisation than human enamel.^24,25^ Human enamel is less dense and has a lower Vickers hardness than bovine enamel, but a higher content of calcium and phosphate ions.^10,15^ In erosion and combined erosion/abrasion experiments, enamel loss was significantly higher in bovine teeth than in human wisdom teeth. No statistically significant difference could be observed in teeth that were only abrasively treated.^5^ However, since the results obtained in the present study were only compared with each other and not with the results of other studies, the use of bovine enamel appears acceptable.

As in several other studies evaluating the erosive potential of acid substances, erosion and abrasion experiments were performed using contact profilometry.^14,37,44^ A fine needle scans the surface profile of the embedded tooth substance and records its height profile. Software calculates the loss of substance by superimposing baseline and post-treatment profiles. The profilometer used in this study has a technical lower detection limit of 0.105 µm.^2^ Damage caused by the profilometer needle itself by sinking into superficially softened enamel layers has been discussed.^35^ The thickness of the superficially softened enamel layer cannot be measured with the profilometer needle and leads to an overestimation of early erosion depth.^35^ However, as only a final profile was recorded (and not between baseline and final), possible damage of the softened enamel surface is insignificant in this study. Contact profilometry has been well validated and is considere a gold standard.^6,36^

A limitation of the study might be that it did not completely reflect the intraoral situation. In this in vitro study, which addressed the demineralising potential of foods/beverages, various individual parameters such as temperature, clearance, buffering capacity of saliva, and the pellicle as protection against erosion were not considered. This is in accordance with other studies, in which the erosive potential of foods/beverages was also assessed.^29,44^ This reproducible experimental setup, however, allowed the investigation of relative differences caused by the tested dressings in relation to orange juice as the control. The main focus was not on the absolute enamel wear of the various bottled salad dressings, but on their relative erosive effects.

Orange juice was used as a control group, as the erosive effects are widely tested and known,^28,46^ and the patients are more aware of the potential erosive effects of orange juice as compared to that of vinegar.

The null hypothesis that there is no statistically significant difference in enamel loss of different bottled salad dressings had to be rejected. All dressings had an acidic pH, but some of the dressings caused statistically significantly higher enamel wear than did orange juice, while others caused statistically significantly lower enamel wear. Compared with orange juice, erosion with balsamic vinegar-based dressings (Italian type) led to statistically significantly greater erosive substance loss. On the other hand, erosion with dressings containing milk and/or cream (French type) caused lower erosive substance loss than Granini orange juice.

Considering the results and the investigated chemical properties of the dressings, it becomes clear that the pH per se does not provide sufficient information about the erosive potential. Although all tested products showed an acidic pH (range: 3.12 to 4.07), statistically significant differences in the erosive potential of the dressings and no increase of erosive/abrasive wear with decreasing pH was observed. The Coop Qualité & Prix French Dressing has a lower pH (3.57) than the orange juice (3.76), but still led to statistically significantly lower enamel wear. Of all the tested products, Tradition Sauce Balsamique is the dressing with the lowest pH (3.12), but with a high calcium content (5.27 mmol/l). It showed lower erosive/abrasive enamel wear than Thomy Balsamico Vinaigrette Dressing with a higher pH (3.64) but less calcium (3.21 mmol/l). This comparison shows that the calcium concentration seems to have a decisive influence on the erosive potential of the dressings in addition to the pH. These findings are consistent with the literature: Not the pH itself, but the degree of saturation of dissolved substances in a liquid at a given pH is essential for demineralisation.^29,39,42^ Particularly the calcium content (and to a lesser extent the fluoride and phosphate content) of the liquid is decisive in determining whether a liquid is saturated with respect to the tooth structure. If the content of the above-mentioned dissolved minerals is too low, the liquid is undersaturated, thus causing demineralisation to establish equilibrium. High concentrations of calcium in a low-pH liquid can consequently counteract erosion, while little or no calcium in a liquid can lead to demineralisation even at a higher pH.^39^ Foods with a naturally high calcium content such as yoghurt or milk show low or no erosive potential despite their low pH.^25,29^ Furthermore, several studies showed that the addition of calcium to acidic beverages can decrease their erosive potential.^7,42,45^

It can be assumed that the difference in erosive potential of Italian Style and French Style dressings seems to be related to the different ingredients and the difference in calcium content. Other factors of the dressings, such as viscosity,^8^ type of acid^38^ and the movement of the liquid on the samples,^3,14^ might also have an impact on the erosivity, but were not considered in this preliminary study.

## Conclusion

Given the findings of the present study and within its limitations, it can be concluded that bottled salad dressings may have erosive potential. Patients with active erosive toothwear or those with high risk should avoid pure balsamico vinegar-based salad dressings and instead choose dressings containing calcium-rich products (enriched with milk and/or cream). These patients should be made aware of the findings so that they can reconsider their purchase decision of bottled dressings. Further studies are needed to develop approaches to how consumers can modify bottled salat dressings to reduce their erosive potential.
